# Hydraulic strategies of *Cunninghamia lanceolata* under drought are shaped by native drought conditions

**DOI:** 10.48130/forres-0025-0031

**Published:** 2025-12-31

**Authors:** Jian Feng, Yuan Yao, Yue He, Pei Wang, Hanying Hu, Sheng Zhang

**Affiliations:** 1 Key Laboratory for Bio-resource and Eco-environment of Ministry of Education, College of Life Sciences, Sichuan University, Chengdu 610065, China; 2 Chengdu Botanical Garden-Sichuan University Joint Laboratory for Ex Situ Conservation and Resource Utilization of Mountain Plants, Sichuan University, Chengdu 610065, China; 3 Sichuan Ecological Protection and Construction Engineering Technology Research Centre, Sichuan University, Chengdu 610065, China

**Keywords:** Chinese fir, Metabolome, Provenance, TVDI, Physiology

## Abstract

*Cunninghamia lanceolata* is integral to soil conservation, climate regulation, and biodiversity maintenance, yet its ecological functions are threatened by drought. Functional trait trade-offs underpin hydraulic safety, but the plasticity of hydraulic strategies in *C. lanceolata* remains poorly understood. Here, the historical intensity of drought across different *C. lanceolata* regions were quantified using the Temperature Vegetation Dryness Index (TVDI) derived from satellite remote sensing. Seedlings sourced from these regions were subjected to drought under greenhouse conditions, and then water status, physiological traits, and metabolic responses were assessed to elucidate hydraulic strategies. The results revealed that TVDI effectively captured regional drought patterns, with the Dechang region experiencing the highest drought intensity, followed by the Hongya and Shiyan regions. The seedlings exhibited distinct hydraulic responses under drought stress. The highest drought-originated seedlings maintained a stable leaf water status, imposed stricter regulation of stomatal and biomass, and accumulated higher levels of antioxidants and defense compounds, indicative of a conservative strategy. In contrast, the low drought-originated seedlings showed greater fluctuations in leaf water content and potential, retained more aboveground biomass, and accumulated fewer defense compounds, reflecting an acquisitive strategy. The moderate drought-originated seedlings adopted an intermediate strategy, balancing growth and antioxidant accumulation. Overall, as the intensity of the drought increased across the provenances, *C. lanceolata* shifted from an acquisitive to a conservative hydraulic strategy. By linking provenance-specific drought regimes with physiological and metabolic responses, this study provides new insights into drought resistance mechanisms and informs species selection and forest management under climate change.

## Introduction

Climate change has led to more frequent and severe droughts, inducing water deficits that cause hydraulic failure in trees, thus significantly inhibiting growth, and reducing survival rates^[1]^. For example, drought events resulted in the death of 102 million trees in California (2012–2015), while heat-drought events in Germany (2018–2019) increased tree mortality rates from 0.25% to 1.73%, considerably affecting regional ecosystem productivity^[2,3]^. Future climate scenario models predict an increase in the frequency of droughts in many regions^[4]^, which will pose significant challenges to tree survival.

Drought is shaped by precipitation, temperature, soil properties, and vegetation cover. Traditional drought indices, such as the Soil Water Index^[5]^, Palmer Drought Severity Index^[6]^, and Standardized Precipitation Index^[7]^, rely primarily on temperature or precipitation, and often fail to capture drought complexity, including plant resilience^[8]^. Integrating spectral indices with meteorological data improves the accuracy of drought assessment. The Temperature Vegetation Dryness Index (TVDI), derived from Land Surface Temperature (LST) and Normalized Difference Vegetation Index (NDVI), effectively evaluates vegetation drought stress^[9]^. Its physical basis and accessibility via remote sensing, make it a valuable tool for monitoring vegetation drought^[9]^. Previous studies have further confirmed its utility across various ecosystems. For instance, Son et al.^[10]^ showed that TVDI was more sensitive than the Crop Water Stress Index in detecting drought events in the Lower Mekong Basin, effectively capturing agricultural drought severity. Similarly, Liu et al.^[11]^ demonstrated that TVDI derived from MODIS data accurately differentiated drought levels across China, providing a reliable tool for large-scale monitoring. Furthermore, Li et al.^[12]^ compared TVDI with other drought indices, including the Synthesized Drought Index, Scaled Drought Condition Index, Vegetation Condition Index, and Temperature Condition Index, and found that TVDI consistently delivered reliable results for monitoring drought across mainland China. These studies underscore the robustness of TVDI in capturing both temporal and spatial variations in drought severity.

Maintaining hydraulic integrity under drought stress is vital for tree survival in water-limited environments^[13]^. Water uptake and transport in trees are governed by various functional traits, including root growth, stomatal regulation, and transpiration^[14]^. This emphasizes that no single functional trait can fully characterize tree survival under drought stress. Instead, integrating morphological, physiological, and biochemical traits is essential to understand the strategies and mechanisms of plant water regulation.

Trees maintain hydraulic safety under drought through trade-offs among functional traits, including prioritizing root growth for water uptake^[15]^, reallocating non-structural carbohydrates to maintain cell turgor^[16]^, and adjusting stomatal closure to minimize water loss while sustaining photosynthesis^[17]^. These trade-offs vary with species traits, life-cycle strategies^[18]^, drought history^[19]^, and drought timing/duration^[20]^. Trees from different provenances exhibit different strategies for water conservation under drought stress. For example, *Balanites aegyptiaca* seedlings from low-precipitation regions significantly reduce leaf area while increasing root biomass, compared to those from high-precipitation regions^[21]^. Similarly, *Pseudotsuga menziesii* from drier sites increases reactive oxygen scavengers to enhance antioxidant defenses, while those from wetter regions synthesize nitrogen-based osmolytes to maintain cellular water potential through osmotic adjustment, a key mechanism that helps plants sustain cell turgor under drought stress^[22]^. These findings highlight the importance of integrating hydraulic and metabolic traits to fully understand provenance-specific adaptations to drought, which is essential for predicting forest resilience in the face of climate change.

*Cunninghamia lanceolata*, a fast-growing evergreen conifer, is extensively cultivated in southern China^[23]^. The *C. lanceolata* plantation plays a crucial role in the local landscape by providing multiple ecosystem services, including soil and water conservation, climate regulation, and biodiversity support^[24]^. Despite relatively high annual precipitation, uneven seasonal rainfall, and increased evaporation have heightened drought risks, threatening forest ecosystems^[24]^. Drought severely impacts the growth and survival of *C. lanceolata*, as it often coincides with the fastest growth phase of the species, specifically in summer and fall^[23]^. Understanding the hydraulic strategies of *C. lanceolata* is essential for sustainable timber production, ecosystem balance, and enhanced ecohydrological functions. Previous studies have shown that *C. lanceolata* provenances from regions with contrasting mean annual precipitation exhibit contrasting drought responses. Gao et al.^[25]^ reported that seedlings from drier regions tolerated drought via enhanced carbon and nitrogen metabolism, accumulation of compatible solutes, and elevated antioxidant activity, whereas those from wetter regions relied primarily on biomass allocation and stomatal regulation. While these findings highlight key physiological and biochemical differences among provenances, how these traits integrate into coordinated hydraulic adjustments remains unclear. Furthermore, classifying provenances solely by mean annual precipitation may not adequately capture the complexity of drought regimes in their native habitats, thereby constraining the ecological interpretation of provenance-specific adaptations.

To address this knowledge gap, MODIS satellite remote sensing data were used to systematically assess the historical drought conditions in three regions where *C. lanceolata* is distributed. Concurrently, *C. lanceolata* samples from these regions were collected and subjected to drought stress experiments. The key physiological and metabolic traits associated with water maintenance were measured to elucidate the hydraulic strategies shaped by the climate of native origin. Two hypotheses were tested: (1) the water regulation capacity of *C. lanceolata* was significantly influenced by the water conditions of the native habitats; (2) under drought stress, trees from drier regions would adopt more conservative hydraulic strategies.

## Materials and methods

### Remote-sensing data sets

MODIS remote sensing data (MOD13Q1 and MOD11A2) from the National Aeronautics and Space Administration (NASA) was used, covering January 2000 to December 2020. MOD13Q1 provides an Enhanced Vegetation Index (EVI) at a resolution of 250 m. The maximum EVI value was extracted monthly and resampled to a uniform 1 km resolution. MOD11A2 provides LST data at a 1 km resolution. The average daytime LST was extracted monthly, corresponding to the MODIS Terra satellite's overpass time of approximately 10:30 a.m. local time. Quality control excluded cloud- and snow-contaminated pixels, and bilinear interpolation filled data gaps. The Savitzky-Golay filter was applied to reduce temporal noise^[26]^. Elevation data from NASA's NASADEM product (30 m resolution) were resampled to 1 km for LST correction and TVDI calculation. All pre-processing and analyses were performed on the Google Earth Engine (GEE) platform.

Numerous studies have demonstrated a strong negative correlation between TVDI and *in situ* soil moisture across diverse ecosystems^[8,27,28]^, validating its use as a proxy for surface water deficit. To evaluate its reliability in the present study region, satellite-derived TVDI values were compared with ground-based volumetric soil moisture measurements obtained from the China Science Data Bank^[29]^. Soil moisture was determined using the gravimetric method. The relationship was quantified using linear regression, with slope, R^2^, and Pearson's r quantifying the strength and direction of the association. Model performance was further evaluated by RMSE, and the statistical significance of the slope was tested using *p*-values. To enhance interpretability, effect sizes (Cohen's f^2^ and *η*^2^) were calculated; and robustness was ensured by bootstrap resampling (1,000 iterations) to generate 95% confidence intervals for the slope. All analyses were performed in R (version 4.3.1) with the 'ggplot2' package (v3.5.2) for visualization, the 'effectsize' package (v1.0.1) for effect size estimation, and the 'boot' package (v1.3-31) for resampling.

### Remote sensing data analysis

To account for the effect of elevation on LST, the following formula, Eq. (1) was applied.

1 $ {{T}}_{{S}}={T}+\mathrm{a}H $

where, *T*_*S*_ is the corrected LST (°C), *T* is the original LST from MOD11A2 (°C), *a* is the correction coefficient set at 0.006^[30]^, and *H* is the elevation (m).

Following the general framework proposed by Sandholt et al.^[31]^, the TVDI was constructed using a two-dimensional feature space defined by LST and NDVI. In this study, the EVI was used instead of NDVI to reduce saturation effects and better represent vegetation structure, particularly in areas with high biomass^[32]^. TVDI was calculated using Eq. (2):

2 $ \mathrm{TVDI}=\dfrac{T_S-T_{min}}{T_{max}-T_{min}} $

where, *T*_*S*_ is the LST of any pixel, *T*_*min*_ is the lower boundary (wet edge) of the LST-EVI, and *T*_*max*_ is the upper boundary (dry edge)^[27]^. TVDI values range from 0 to 1, with higher values indicating more severe drought and lower values reflecting wetter conditions. The wet and dry edges were determined by linear regression and expressed as:

3 $ T_{min}=\mathrm{\mathit{a}}_1+\mathit{\mathrm{\mathit{b}}_{\mathrm{1}}}EVI $

4 $ T_{max}=\mathrm{\mathit{a}}_2+\mathrm{\mathit{b}}_2\cdot EVI $

where, *a*_1_ and *a*_2_ are intercepts, and *b*_1_ and *b*_2_ are slopes of the wet (Eq. [3]), and dry (Eq. [4]) edge equations, respectively. These parameters were derived using least squares regression by extracting the minimum and maximum LST values within EVI intervals of 0.01 across the LST–EVI feature space.

To analyze temporal trends in drought conditions, the Theil-Sen median method was used to calculate trends in monthly mean TVDI values, and the Mann-Kendall test was used to assess the significance of the trend^[33]^. All calculations were performed using Python (version 3.10) and figures were generated with ArcGIS 10.8 (Esri, Redlands, CA, USA). Further details on the collection, processing, and computation of remote sensing data can be found in Supplementary File 1.

### Plant materials, experimental design, and sampling strategies

**Figure 1:**
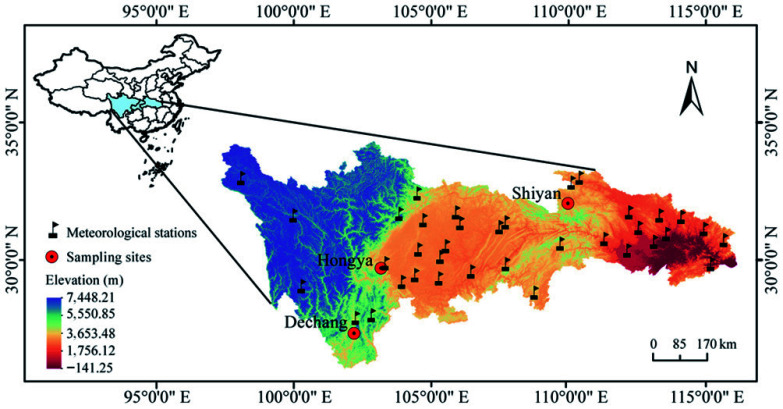
The spatial distribution of the sampling sites and the 36 meteorological stations selected for this study. The scale bars in the panels represent a horizontal ground distance of 170 km.

The experimental materials comprised two-year-old *C. lanceolata* seedlings, sourced from seeds of locally adapted populations with a long history of cultivation across three provenances: Shiyan, Dechang, and Hongya. Based on long-term drought patterns derived from the TVDI, Shiyan was classified as the lowest drought-intensity region (LDR), Hongya as moderate (MDR), and Dechang as the highest (HDR). The environmental conditions of these habitats summarized are detailed in Table 1 and Fig. 1. In May 2020, seedlings exhibiting similar growth characteristics were selected to minimize the influence of non-drought factors.

**Table 1:** Environmental characteristics of the three *C. lanceolata* provenances.

Provenance	Area	Latitude	Longitude	Altitude (m)	MAT (°C)	MAP (mm)
Dechang	Dechang County, Sichuan province, China	27.34	102.19	1,407.13	17.70	1,074.40
Hongya	Hongya County, Sichuan province, China	29.70	103.18	1,244.88	16.90	1,362.80
Shiyan	Shiyan City, Hubei province, China	32.06	109.97	947.09	15.40	769.60
Data on mean annual temperature (MAT) and mean annual precipitation (MAP) were obtained from local government archives, which compile long-term meteorological records and are widely used in regional studies. Although the archives do not explicitly specify the temporal coverage, these values provide a reliable representation of local climate conditions. Elevation data were obtained from the NASADEM Digital Elevation Model at a 30 m resolution.						

The selected seedlings, averaging 45 cm in height and approximately 5 mm in basal stem diameter, were transplanted into a greenhouse at the experimental station in Chengdu, China. Each seedling was planted individually in a pot containing 40 l of homogeneous substrate (nutrient soil : vermiculite = 1:1), characterized by low bulk density and high porosity. In the control group (CK), substrate moisture was maintained at approximately 50% volumetric water content, which approximates the field capacity of this substrate mixture and thus represents well-watered conditions. Greenhouse conditions were maintained at 23 °C, 70% relative humidity, and a 12-h photoperiod. The light period began at 06:00 and ended at 18:00. The primary objective of the experiment was to assess the plastic responses of seedlings from different provenances to drought stress, with a particular focus on how local adaptation influences their ability to cope with water stress.

The experimental design, including drought treatments and seedling management, followed the methods outlined by Gao et al^[25]^. The seedlings were acclimatized for two months, during which the soil moisture content in each pot was measured using a portable soil moisture meter (Field Scout TDR 350, Spectrum Technologies, Inc., USA) to ensure that the soil volumetric water content remained at 50%. Subsequent to the acclimation period in July 2020, 30 healthy, disease-free seedlings with similar growth were selected from each provenance, making a total of 90 seedlings for the experiment. The seedlings of each provenance were randomly divided into two groups for a two-factor experiment involving provenance and drought conditions. A group served as the control (CK), receiving normal watering to maintain the volumetric water content of the soil at 50%. The other group was subjected to drought treatment (D) with reduced watering. The drought treatment lasted 40 d, with the first 10 d reducing the volumetric water content of the soil from 50% to 20%. For the next 30 d, the volumetric water content of the soil was maintained at 20% through controlled watering. Soil moisture content was measured daily at 09:00 and 17:00 using a portable soil moisture meter to ensure that soil moisture remained within the set range, with additional watering provided when necessary.

After 30 d of drought treatment, 10 seedlings were randomly selected from each provenance within each treatment group. Mature leaves were collected between 12:00 and 13:00 from the third fully expanded branch counted from the top, which was consistently exposed to light and free from shading, to ensure physiological uniformity. For each provenance under each treatment, three leaves were collected from each seedling and then pooled. The leaves were evenly divided into three portions by fresh weight, with each portion serving as an independent biological replicate for subsequent analyses. One portion was immediately used to measure leaf water potential and relative water content, while the remaining portions were flash-frozen in liquid nitrogen and stored at −80 °C for subsequent metabolite analysis.

### Measurements of leaf water status

After leaf collection at noon, leaf water potential was immediately measured using a WP4C Dewpoint Potentiometer (WP4C, METER Group, Inc., Pullman, WA, USA). Leaf relative water content (RWC) was calculated using Eq. (5):

5 $ {RWC}=\dfrac{{FW-DW}}{{TW-DW}}{100{\%} } $

where, *FW* is the fresh weight, *TW* is the turgid weight, and *DW* is the dry weight. *FW* was measured immediately after sampling. *TW* was obtained by soaking leaves in deionized water for 12 h in darkness at room temperature, and then blotting them dry with filter paper. *DW* was determined by oven-drying the leaves at 80 °C for 72 h until a constant weight was achieved. The leaf samples were measured using a high-precision analytical balance (ME204, Mettler Toledo, Switzerland).

### Recalculation of leaf physiological indices

A previous study had reported that *C. lanceolata* from three provenances exhibited differential physiological responses to drought stress^[25]^. However, it did not fully account for the inherent physiological differences among provenances or establish a direct relationship between these differences and the severity of drought in their native regions. This limitation may hinder a comprehensive understanding of drought adaptation strategies of *C. lanceolata* and highlights the need for more precise evaluation of drought resistance. To address these limitations, the original physiological dataset from that study was reanalyzed in the present work. A broader set of metrics were incorporated, including biomass indices: leaf weight (LW), stem weight (SW), aboveground biomass (AB), root weight (RW), root-to-shoot ratio (RST), and total weight (TW); photosynthetic parameters: net photosynthesis rate (Pn), stomatal conductance (Gs), intercellular CO_2_ concentration (Ci), transpiration rate (Tr), and instantaneous water use efficiency (WUEi); antioxidant enzymes: peroxidase (POD), superoxide dismutase (SOD) and ascorbate peroxidase (APX); and osmotic adjustment compounds: proline (Pro), soluble sugars (SS), sucrose (SU), levulose (LE), starch (ST) and nonstructural carbohydrates (NSC). Detailed measurements of the indicators are provided in Supplementary File1, section 3. In addition, two physiological indicators, namely relative water content and water potential, were newly measured in the current experiment and included in the analysis. To minimize the influence of inherent differences among provenances and better isolate the drought effects, the percentage change for each trait was calculated using Eq. (6), as follows:

6 $ {\mathrm{Percentage}\;{\rm change}}=\dfrac{(D\; value-CK\; value)}{CK\; value}{100{\%} } $

where, *CK* represents the control, and *D* represents the drought treatment.

### Measurement of leaf metabolites

Leaf metabolites of *C. lanceolata* subjected to various treatments were quantified using a broad-targeted metabolomics approach. The freeze-dried leaf samples were ground using a ball mill (MM 400, Retsch, SCIENTZ, China), extracted with 70% methanol, and centrifuged prior to UPLC-MS/MS analysis. Chromatographic separation was performed on a Shimpack UFLC SHIMADZU CBM30A system (Shimadzu Corporation, Japan) with an Agilent SB-C18 column, using a gradient solvent system of solvent A (0.1% formic acid + 99.99% ultrapure water), and solvent B (100% acetonitrile, Thermo Fisher Scientific, USA). The detection of MS/MS was carried out on an Applied Biosystems 4500 QTRAP (AB SCIEX, USA) with electrospray ionization (source temperature 550 °C, ion spray voltage 5,500 V, curtain gas 30 psi). Metabolites were identified by comparing m/z values, retention times, and fragmentation patterns with the MWDB database (Metware Biotechnology Co., Ltd., Wuhan, China), with data processed using Analyst (version 1.6.3), and MultiaQuant software (SCIEX, USA). See Supplementary File1 for more details.

### Statistical analysis

The normality and homogeneity of variances for all datasets were checked and log_2_-transformed when necessary before analysis. Analysis of variance (ANOVA) was performed using the 'aov' function in R (version 4.3.1) for relative water content and water potential, followed by Tukey's honestly significant difference (HSD) test using the 'TukeyHSD' function in R for post hoc comparisons. Independent *t*-tests were performed with the 't.test' function in R to assess the significance of relative changes in the various indicators. A consistent significance threshold of *p* < 0.05 was applied across all statistical tests, including *t*-tests, ANOVA, and Tukey's HSD. Principal component analysis (PCA) was conducted using the 'FactoMineR' package (version 2.11) in R, and data visualization was performed with the 'pheatmap' (version 1.0.12), and 'ggplot2' packages (version 3.5.2). Hierarchical clustering analysis (HCA) was conducted using the 'pheatmap' package, applying Euclidean distance and complete linkage to explore provenance-specific patterns in metabolite profiles. The 'ropls' package (version 1.36) was used for orthogonal partial least squares discriminant analysis (OPLS-DA) of metabolomic data. Significantly differential metabolites (DEMs) were identified based on the fold change (FC) values and the VIP scores from the OPLS-DA model (|log_2_ FC| ≥ 1 and VIP ≥ 1). To statistically test group differences, permutational multivariate analysis of variance (PERMANOVA) was applied to the Euclidean distance matrix derived from the original data using the vegan package (version 2.6). Kyoto Encyclopedia of Genes and Genomes (KEGG) pathway enrichment analysis was performed using the KEGG database (https://www.kegg.jp/), with *Populus trichocarpa* as the reference background^[34]^. Pathways with *p* < 0.05 were considered significantly enriched.

## Results

### Characteristics and trends of drought in different provenance regions

**Figure 2:**
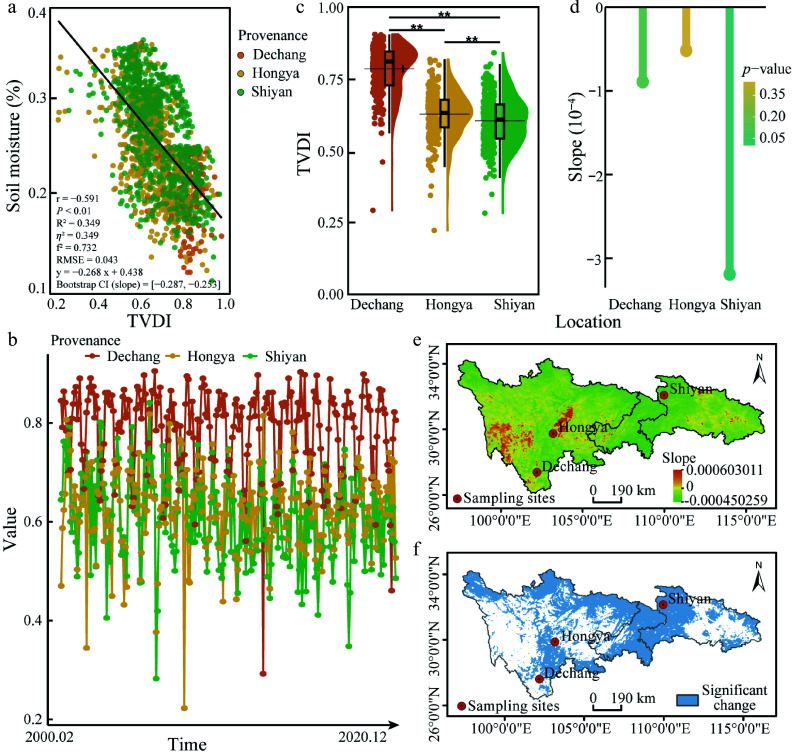
Validation of TVDI results and its variation across the three provenance regions. (a) Correlation between TVDI and soil moisture. Each data point represents a meteorological station, and colors are assigned based on the Euclidean distance to the three sampling points. The color indicates the nearest sampling point to each meteorological station. The black solid line represents the linear regression trend for all the data. (b) Monthly TVDI of the three provenances from February 2000–December 2020. (c) Annual mean TVDI from 2000 to 2020; (d) Trends and significance of TVDI changes across the three provenance regions from 2000 to 2020. (e) Theil-Sen slope estimates of TVDI in the study area; (f) Mann-Kendall test for the significance of TVDI trends in the study area. The scale bars in these panels represent a horizontal ground distance of 190 km.

This study validated the reliability of TVDI using soil moisture measurements at a depth of 20 cm. After removing invalid data, 36 meteorological stations with usable records were included in the analysis (Fig. 1). A significant negative correlation was observed between TVDI and soil moisture (r = −0.591; *p* < 0.01) (Fig. 2a). The model explained 34.9% of the variance (R^2^ = 0.349) and demonstrated good predictive performance (RMSE = 0.043). Effect sizes further indicated that a strong influence of TVDI on soil moisture (Cohen's f^2^ = 0.732; *η*^2^ = 0.349). Bootstrap resampling with 1,000 iterations yielded a 95% confidence interval for the slope (–0.286 to –0.251), confirming the robustness of this relationship. These results collectively support TVDI as a reliable metric for assessing regional drought conditions.

Analysis of TVDI from 2000 to 2020 revealed pronounced spatial variation in drought intensity across the three provenance regions (Fig. 2b). Dechang exhibited the highest annual mean TVDI (0.79), indicating the most severe drought conditions, followed by Hongya (0.63) and Shiyan (0.60), which experienced the mildest drought stress (*p* < 0.01) (Fig. 2c). Over the 21-year period, TVDI exhibited a slight downward trend in all regions. The decline was most pronounced in Shiyan (−3.2 × 10^−4^ yr^−1^; *p* < 0.01), followed by Dechang (−0.89 × 10^−4^ yr^−1^), and Hongya (−0.52 × 10^−4^ yr^−1^) (Fig. 2d–f). Despite these trends, the relatively modest rates of change suggest that long-term drought conditions were largely stable. Together, these results support the classification of Dechang is HDR, Hongya is MDR, and Shiyan is LDR. It provides a consistent ecological framework for subsequent comparisons of drought responses among *C. lanceolata* provenances throughout the remainder of the paper.

### Water status and physiological responses of *C. lanceolata* under drought stress

**Figure 3:**
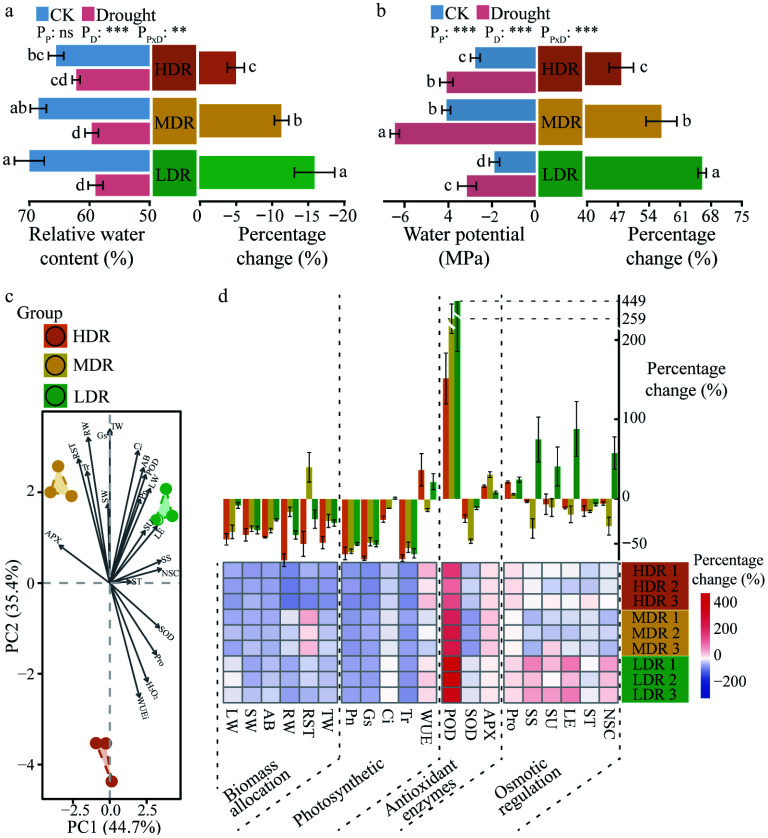
Water status and physiological changes in different provenances of *C. lanceolata* under drought stress. (a) Relative water content and relative changes in leaves of different *C. lanceolata* under drought stress; Provenances are color-coded: orange, HDR; yellow, MDR; green, LDR. (b) Water potential and relative changes in leaves of different *C. lanceolata* under drought stress. The values were means ± SD. Different lowercase letters on the bars represent significant differences (*p* < 0.05) based on ANOVA with Tukey's honestly significant difference test. P_P_, provenance effect; P_D_, drought effect; P_P × D_, provenance × drought interaction. (c) PCA of relative changes in physiological traits of *C. lanceolata* from the three provenance regions; (d) Relative changes in physiological traits of *C. lanceolata* from three provenance regions under drought stress. The bar plot (top) shows the mean percentage change of each physiological trait for each provenance under drought stress, expressed as mean ± SD. The heatmap (bottom) displays the percentage change of each trait across three biological replicates per provenance, highlighting individual-level variability. The abbreviations are provided in Section 'Recalculation of leaf physiological indices'.

The water status of *C. lanceolata* leaves varied significantly among the three provenance regions under drought stress. After drought treatment, relative water content and water potential decreased significantly in seedlings from LDR and MDR (*p* < 0.05) (Fig. 3a, b). In contrast, HDR seedlings showed a significant reduction only in water potential, while relative water content remained statistically unchanged (Fig. 3a, b). In terms of relative changes, HDR seedlings experienced smaller reductions in leaf relative water content (5.10%), and water potential (48.21%) compared to MDR (11.36% and 57.27%), and LDR seedlings (15.97% and 66.44%) (*p* < 0.05) (Fig. 3a, b), suggesting better water status maintenance in HDR seedlings under drought stress.

The PCA results revealed distinct regional differences in physiological responses among the three *C. lanceolata* provenances (Fig. 3c). The two principal components together explained 80.1% of the total variance. The first principal component separated HDR and MDR from LDR, primarily driven by variations in ascorbate peroxidase, superoxide dismutase, nonstructural carbohydrates, and soluble sugars, which reflect differences in antioxidant capacity and osmotic adjustment. The second principal component separated HDR from MDR and LDR, mainly due to differences in total weight, stomatal conductance, intercellular CO_2_ concentration, and instantaneous water use efficiency, reflecting variations in biomass allocation and photosynthetic performance among the provenances (Fig. 3c). PERMANOVA performed on the original Euclidean distance matrix independently confirmed that these differences were statistically significant (R^2^ = 0.98; *p* = 0.004). HDR seedlings showed a 65.51% decrease in stomatal conductance, a significantly greater reduction than that observed in MDR seedlings (47.30%) and LDR seedlings (49.65%) (*p* < 0.01). A similar trend was observed in intercellular CO_2_ concentration (Fig. 3d; Supplementary Tables S1, S2). For biomass allocation, MDR seedlings exhibited the smallest decrease in root weight and a 40.66% increase in the root-shoot ratio, while HDR (57.51%) and LDR (26.55%) seedlings decreased more (*p* < 0.01) (Fig. 3d; Supplementary Tables S1, S2). Interestingly, HDR seedlings experienced a 49.13% reduction in aboveground biomass, significantly greater than MDR seedlings (40.47%) and LDR seedlings (26.66%) (*p* < 0.05) (Fig. 3d; Supplementary Tables S1, S2). In LDR seedlings, the contents of soluble sugar, sucrose, levulose, and nonstructural carbohydrates increased by 64.16%, 35.12%, 75.47%, and 43.50%, respectively, while they decreased in HDR and MDR seedlings (Fig. 3d; Supplementary Table S1). Additionally, antioxidant accumulation varied among the three provenances (Fig. 3d; Supplementary Table S1).

### Effects of drought on *C. lanceolata* metabolites

**Figure 4:**
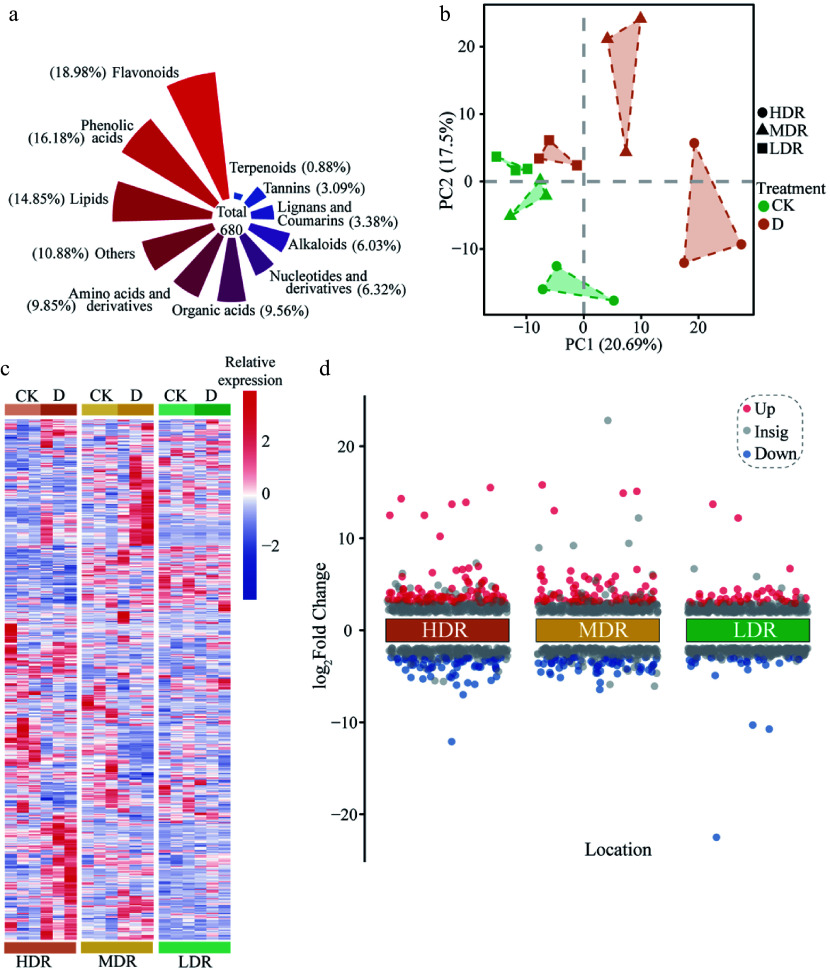
Metabolomics overview and PCA analysis of *C. lanceolata* from different provenance regions under drought stress. (a) Proportion of each metabolite. (b) PCA of all detected metabolites. (c) Hierarchical cluster analysis and heatmap of all metabolites detected in plant leaves from each treatment. CK, Control treatment. D, Drought treatment. (d) Significantly upregulated and downregulated metabolites in *C. lanceolata* leaves. Red represents significantly upregulated metabolites, while blue represents significantly downregulated metabolites.

To further investigate the mechanisms underlying the responses of *C. lanceolata* to drought stress, UPLC-MS/MS was used to analyze leaf metabolites under different treatments. A total of 680 metabolites were identified (Fig. 4a). PCA revealed clear separation among provenances, indicating distinct drought-induced metabolic profiles (Fig. 4b), while hierarchical clustering further emphasized region-specific patterns (Fig. 4c). DEMs analysis (|log_2_ FC| ≥ 1 and VIP ≥ 1) showed the strongest metabolic shifts in HDR seedlings (108 upregulated, 72 downregulated), followed by MDR (87 upregulated, 65 downregulated), and LDR (51 upregulated, 30 downregulated) seedlings (Fig. 4d; Supplementary Table S3).

### KEGG pathway enrichment analysis

**Figure 5:**
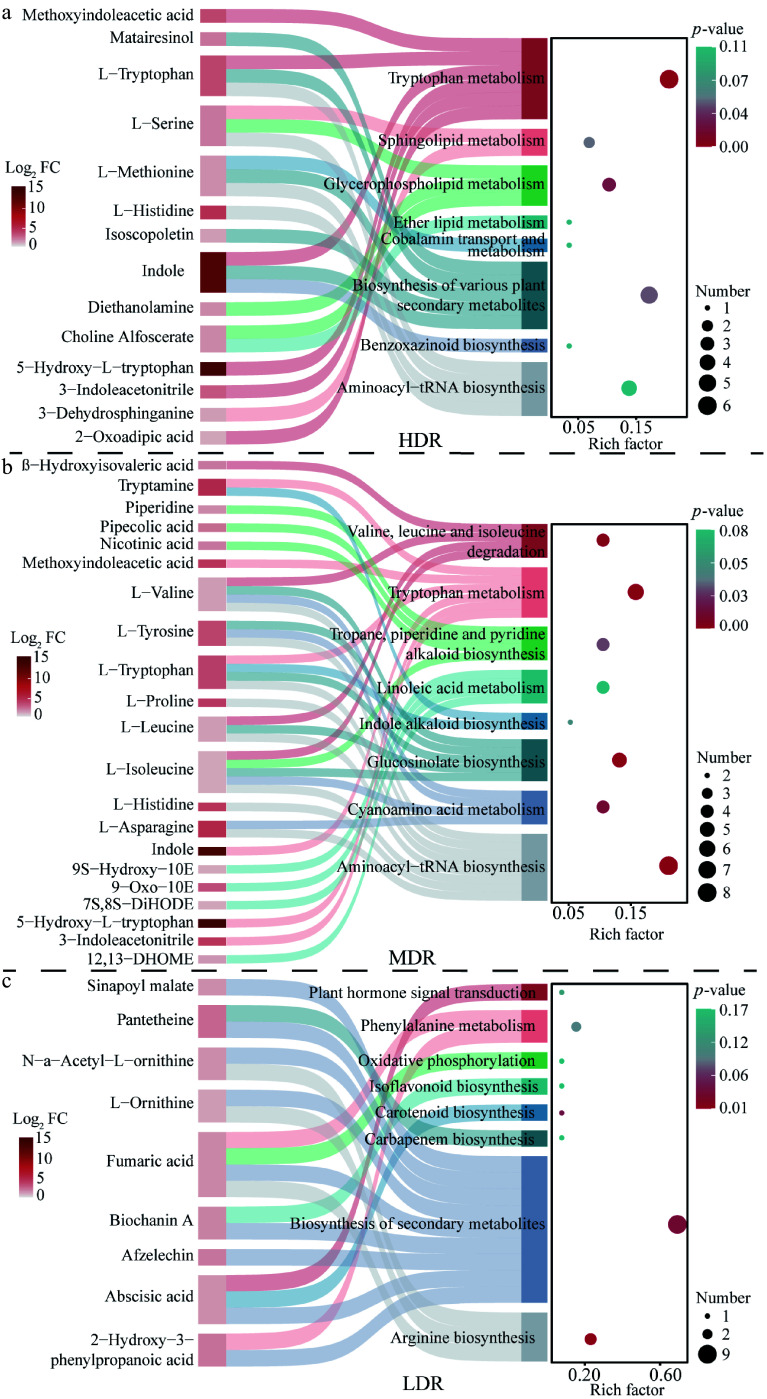
KEGG enrichment analysis of significantly upregulated metabolites in *C. lanceolata* from different provenance regions. The figure combines a KEGG enrichment bubble plot and a Sankey diagram. In the bubble plot, the horizontal axis shows the Enrich factor (number of differentially abundant metabolites mapped to a pathway divided by total metabolites in that pathway), and the vertical axis lists pathway names. Bubble size represents the number of upregulated metabolites, and bubble color indicates the adjusted *p*-value (red to blue: higher to lower significance). The Sankey diagram shows connections between enriched pathways (right) and corresponding metabolites (left); pathway colors are manually assigned for distinction, and metabolite node color represents log_2_ fold change (Log_2_ FC) between drought and control groups. Flow width reflects the number of metabolite-pathway associations.

To assess the involvement of DEMs in metabolic pathways, KEGG enrichment analysis was performed for upregulated DEMs. HDR and MDR seedlings showed 58 enriched pathways (three and seven significantly enriched, respectively), whereas LDR had 32 (three significantly enriched) (Fig. 5; Supplementary Table S4). The tryptophan metabolism pathway was significantly enriched in HDR and MDR seedlings. Notably, 5-hydroxy-L-tryptophan was consistently upregulated across all provenances (Supplementary Table S3). HDR and MDR seedlings also showed elevated levels of indole and L-tryptophan (Fig. 5), indicating enhanced activation of tryptophan metabolism under drought. In contrast, MDR seedlings accumulated multiple branched-chain amino acids, including L-isoleucine, L-leucine, and L-valine, while LDR seedlings were characterized by increased levels of L-ornithine and N-*α*-acetyl-L-ornithine (Fig. 5). These findings highlight distinct provenance-specific metabolic adjustments to drought stress.

### Metabolic network analysis

**Figure 6:**
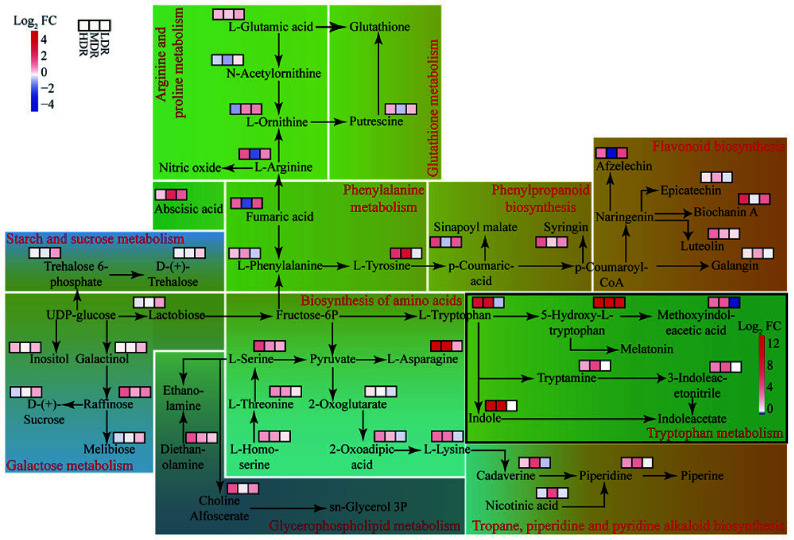
Metabolic pathways of DEMs in *C. lanceolata* provenances under drought stress. FC (fold change) represents the ratio of metabolite abundance under drought vs control, and Log_2_ FC is the log-transformed value. Red indicates higher abundance under drought, while blue indicates lower abundance. Each rectangle represents a metabolic pathway, and the red text along the border indicates the pathway name. Heatmap cells within each pathway show relative changes of individual DEMs across provenances. Cells in black-framed rectangles use a pathway-specific Log_2_ FC color scale; other rectangles use the color scale shown in the top-left corner. Background colors are used to group pathways with similar physiological functions for visual clarity.

Using the KEGG database, a metabolic network to visualize DEMs associations under drought stress was constructed (Fig. 6). Each population exhibited distinct metabolic changes, although some common trends emerged. In HDR seedlings, fumaric acid influenced the urea cycle, thereby increasing L-arginine and affecting nitric oxide levels, whereas a similar, but statistically insignificant trend, was observed in LDR seedlings (Supplementary Table S3). Meanwhile, L-homoserine specifically contributed to L-serine production in HDR seedlings, which in turn affected sn-glycerol-3P metabolism and ethanolamine levels. Furthermore, in HDR and MDR seedlings, fructose-6P and L-homoserine boosted L-asparagine and L-lysine, whereas the downstream effect of L-lysine improving piperidine and piperine synthesis was specific to MDR seedlings. In LDR seedlings, UDP-glucose drove sugar metabolism, accumulating inositol, melibiose, D-(+)-sucrose, trehalose 6-phosphate, and D-(+)-trehalose. Additionally, L-phenylalanine participated in the biosynthesis of phenylpropanoid and flavonoid, leading to the accumulation of sinapoyl malate, syringin, and biochanin A, with variations among the three populations. Fructose-6P influenced indole acetate, with HDR and MDR seedlings showing similar DEMs patterns, distinct from LDR seedlings. Abscisic acid (ABA) levels increased in all provenances, with significance in MDR and LDR seedlings.

## Discussion

### Drought characteristics of the provenance site

Drought significantly affects soil moisture and vegetation growth, with increasing frequency and intensity under climate change, raising concerns^[35]^. Understanding its influence on plant hydraulic strategies is crucial for ecological conservation and resource management. Analysis of average TVDI values over 21 years revealed significant regional differences: Dechang region exhibited the highest values, Shiyan region the lowest, and Hongya region was moderate. These patterns did not fully align with mean annual precipitation patterns, highlighting the limitation of precipitation-based assessments. While precipitation is a commonly used index for drought evaluation, it primarily reflects surface water input and neglects the influence of evaporation, temperature, soil properties, and other factors on actual moisture availability^[36,37]^. Particularly, the mean annual precipitation index fails to account for precipitation intensity and distribution, both of which play crucial roles in shaping local drought conditions. The TVDI results reveal spatial heterogeneity in drought conditions among the three provenance sites, implying distinct adaptive pressures faced by *C. lanceolata* in each region. Further trend analysis indicates that, despite interannual variability, a relatively stable long-term drought pattern persists, imposing consistent selective pressure that may have driven the evolution of divergent hydraulic strategies across populations^[13]^.

Based on these long-term TVDI patterns, all three provenance sites experience persistent drought stress, reflecting sustained water limitation in the field despite regional differences in intensity. Taking into account the water-holding properties of the 1:1 nutrient soil–vermiculite medium used in this study, seedlings from all provenances were subjected in the greenhouse to a standardized soil moisture gradient: 50% volumetric water content served as the control, representing non-stress conditions with adequate water availability, while 20% volumetric water content was applied as the drought treatment to simulate low soil moisture during dry periods. However, due to differences in substrate properties, this treatment does not directly correspond to field soil moisture conditions. This design ensures comparability across provenances under controlled conditions while simultaneously reflecting the water limitations associated with persistent drought stress, and provides a basis to examine provenance-specific hydraulic strategies and drought adaptations in *C. lanceolata*.

### Responses of *C. lanceolata* seedlings to drought

Greenhouse control experiments confirmed our hypotheses, revealing distinct drought responses among the *C. lanceolata* provenances. Although all seedlings exhibited reductions in leaf relative water content and water potential under drought treatment, HDR seedlings maintained the highest absolute RWC compared to MDR and LDR. Importantly, HDR seedlings exhibited the smallest reductions in both relative water content and water potential, indicative of a conservative hydraulic strategy that enhances the maintenance of water status under drought stress. On the contrary, seedlings from the LDR showed the highest variability, suggesting a more plastic response to water stress. These findings support our first hypothesis. This phenomenon also has been observed in other species, including *Pseudotsuga menziesii*^[38]^, *Quercus faginea*^[39]^, and *Pinus pinea*^[40]^. The trends in the regulation of water status reflect both immediate physiological responses and long-term adaptive strategies shaped by native drought conditions. These results underscore the role of local adaptation in shaping hydraulic strategies, and highlight the importance of considering native environments when assessing plant responses to climate change^[41]^.

Phenotypic plasticity in hydraulic traits allows plants to adapt to environmental fluctuations^[1]^. This study reveals significant regional variations in the physiological responses of *C. lanceolata* seedlings to drought stress, influenced by the historical drought conditions of their native regions. Seedlings from HDR exhibited the greatest reductions in aboveground biomass and stomatal conductance, reflecting a conservative hydraulic strategy characterized by strict water regulation and reduced transpiration under stress. Such adjustments in growth and gas exchange help maintain stable leaf water status and improve water-use efficiency, consistent with previous observations that plants from drier environments often adopt conservative strategies to minimize water loss^[42]^. In contrast, seedlings from LDR exhibited greater phenotypic plasticity by accumulating high levels of osmotic regulators, such as proline and soluble sugars. These compounds are crucial for osmotic regulation and protein stabilization under stress^[43,44]^. Similar mechanisms have been shown to improve water retention in *Pinus massoniana*^[45]^. The relatively minimal decline in aboveground biomass from LDR seedlings suggests that they adopt an acquisitive strategy, prioritizing water retention and cellular integrity over significant reductions in growth. On the other hand, seedlings from MDR exhibited more pronounced morphological adjustments, such as the smallest decrease in root biomass and the largest increase in root-to-shoot ratio. Enhanced root allocation is a typical drought response, optimizing water acquisition while balancing between growth and conservation^[46]^. Through adjustments in biomass allocation, MDR seedlings optimized both water acquisition and retention. This study highlights the significance of incorporating provenance-specific drought regimes when evaluating plant responses to climate-induced stress. By linking physiological traits with regional drought histories, this study offers novel insights into the drought adaptation mechanisms of *C. lanceolata*.

Metabolic adaptation, along with physiological plasticity, is essential for plant survival in rapidly changing environments. Environmental pressures disrupt metabolic balance, requiring reconfiguration of metabolic networks to maintain function^[47]^. In this study, a sequential decrease in DEMs in *C. lanceolata* from HDR to MDR and LDR was observed, consistent with the native drought conditions of these provenances. Trees originating from drier regions exhibited more extensive metabolic adjustments under drought stress, indicating stronger reconfiguration of metabolic networks. Notably, 5-hydroxy-L-tryptophan was consistently upregulated across all provenances, suggesting its role as a core drought-responsive metabolite. This highlights that, while the overall extent of metabolic reprogramming varied with provenance, certain metabolites represent conserved components of the drought response in *C. lanceolata*.

Mapping DEMs onto metabolic networks revealed provenance-specific metabolic features. HDR seedlings showed a pronounced increase in L-arginine, a precursor of nitric oxide that promotes stomatal closure and contributes to their conservative water-use strategy^[48]^. By contrast, LDR seedlings exhibited only a slight, non-significant increase in L-arginine, but showed enhanced levels of UDP-glucose through galactose and starch/sucrose metabolism, promoting the synthesis of soluble sugars and polyols for osmoregulation^[49]^. LDR seedlings also accumulated L-ornithine, a precursor of proline, which further contributes to osmotic adjustment under drought. By maintaining turgor pressure, this adjustment supports continued growth under moderate water deficit, representing an acquisitive strategy. Meanwhile, both HDR and MDR seedlings showed marked increases in L-asparagine, a nitrogen storage and transport molecule that releases nitrogen for amino acid and protein synthesis^[50]^. In HDR seedlings, this is beneficial for broader metabolic activities, including the synthesis of antioxidants and protective compounds, thereby reinforcing their conservative hydraulic strategy. In MDR seedlings, the increase in L-asparagine, combined with the accumulation of branched-chain amino acids, including L-isoleucine, L-leucine, and L-valine, may enhance nitrogen remobilization and provide additional substrates for protein synthesis, facilitating root growth and biomass maintenance, thereby buffering hydraulic responses and supporting intermediate flexibility. Hormonal regulation also played a crucial role in the response to drought. ABA levels increased across all provenances, triggering stomatal closure, regulating growth, and promoting the accumulation of osmoprotectants^[51]^. Additionally, tryptophan metabolism was enriched in HDR and MDR seedlings, with increased levels of indole and 5-hydroxy-L-tryptophan potentially enhancing auxin synthesis. Auxins act synergistically with ABA to reduce stomatal density, promote root elongation, and reallocate assimilates^[52,53]^, thereby reinforcing water conservation strategies in drier provenances. Antioxidant defenses played a key role in drought adaptation. All provenances exhibited significant yet variable accumulation of antioxidant enzymes following drought. Metabolic analyses further revealed that HDR seedlings possessed a broader spectrum of non-enzymatic antioxidants, including diverse phenolic acids, flavonoids, and alkaloids; LDR seedlings exhibited a narrower range; and MDR seedlings showed an intermediate pattern. This provenance-specific divergence underscores differential regulation of antioxidant defenses. These compounds function to eliminate reactive oxygen species and maintain cellular redox balance, thereby ensuring cell survival under prolonged water deficit^[54]^. Furthermore, HDR seedlings showed pronounced accumulation of diethanolamine and choline alfoscerate, key intermediates in phospholipid metabolism that support cell membrane integrity and stability under drought stress^[55]^. In contrast, both MDR and LDR seedlings exhibited only slight, non-significant increases, indicating that membrane-protective metabolic adjustments were most pronounced in HDR seedlings. Overall, these metabolic adjustments aligned with physiological responses, highlighting distinct metabolic characteristics associated with hydraulic responses in different populations of *C. lanceolata*.

### Hydraulic strategies shaped by native drought conditions

**Figure 7:**
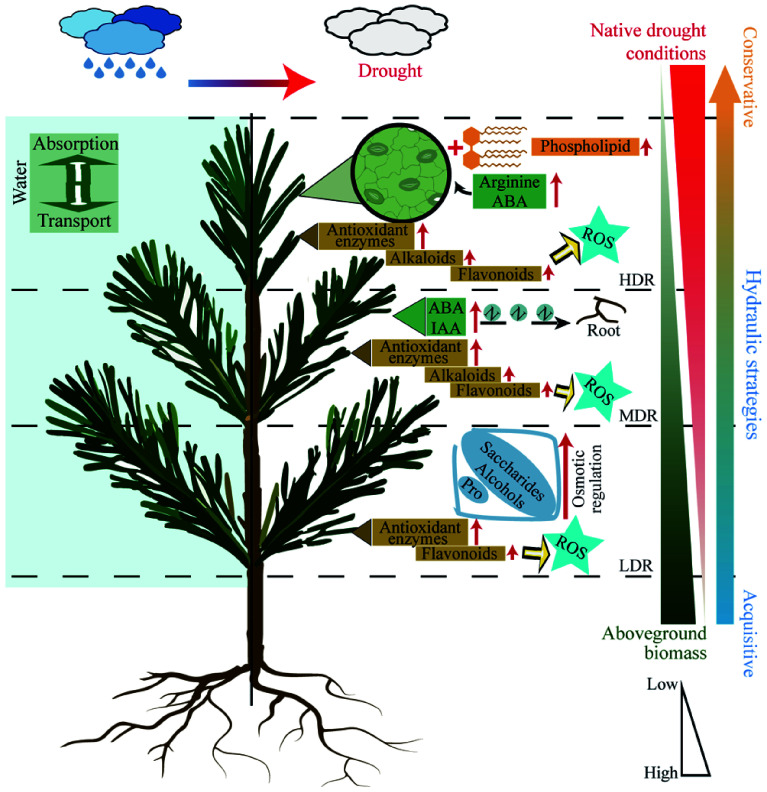
Schematic representation of drought responses in *C. lanceolata* from different provenances. Different dashed lines represent different provenances. The red arrows along the text indicate an increase in content, and yellow arrows indicate direction. ABA, Abscisic acid. ROS, Reactive oxygen species. IAA, Auxin. Pro, Proline.

Plants exhibit considerable variation in hydraulic strategies. Some species favor rapid growth through frequent water use, whereas others adopt more conservative approaches that enhance drought tolerance^[56,57]^. Under drought stress, *C. lanceolata* seedlings showed a clear gradient in water status fluctuations that was negatively associated with the severity of drought in their native regions, with individuals from more arid areas exhibiting smaller variations (Fig. 7). When physiological and metabolic responses were considered together, seedlings from drier regions not only maintained more stable water status but also exhibited significantly reduced aboveground biomass and greater accumulation of key metabolites, such as antioxidants and osmolytes. Long-term adaptation to drought often results in conservative resource use, where plants reduce growth to enhance drought tolerance^[47]^. For example, oaks in warm temperate regions reduce aboveground growth under prolonged drought to maintain reserves of non-structural carbohydrates and sustain drought-resistance metabolism^[58]^. Based on the trade-offs of functional traits observed in different *C. lanceolata* provenances under drought stress, HDR seedlings exemplified a conservative hydraulic strategy. They restricted water loss through stricter stomatal regulation, thereby avoiding catastrophic hydraulic failure^[59]^. The combination of reduced aboveground biomass and elevated antioxidant accumulation further alleviated survival stress. In contrast, LDR seedlings employed an acquisitive strategy, accumulating osmotic substances to facilitate water transport and sustain biomass growth above ground. Their lower antioxidant metabolism suggests a growth-oriented trade-off rather than a tolerance strategy. Meanwhile, MDR seedlings adopted a balanced strategy, maintaining growth while accumulating antioxidants. Their significant increase in the root-to-shoot ratio improved water absorption, while antioxidants mitigated cellular damage^[54]^. Overall, with increasing drought severity in the native habitat, *C. lanceolata* provenances exhibited a shift from an acquisitive to a conservative water-use strategy under drought stress, reflecting adaptive trade-offs and physiological plasticity in response to water limitation.

This study builds upon and extends the work of Gao et al.^[25]^ by providing a more integrated understanding of the hydraulic strategies in *C. lanceolata* seedlings from different provenances in response to drought. While Gao et al.^[25]^ relied on precipitation-based drought classifications, this research utilizes long-term remote sensing data, enabling a more detailed and accurate assessment of drought severity across *C. lanceolata* source regions. By applying the TVDI, which integrates temperature and vegetation dynamics, this study offers a more comprehensive characterization of the drought conditions shaping plant hydraulic responses and adaptation, thereby overcoming the limitations of traditional approaches based on single meteorological indicators. Furthermore, this study introduces a novel metabolic-level perspective, investigating the physiological and biochemical traits that govern the hydraulic strategies employed by *C. lanceolata* under drought stress. Although Gao et al.^[25]^ identified general trends in the effects of drought on growth and physiological traits, this research further elucidates how distinct metabolic responses—such as osmotic regulation and antioxidant activity—underpin the differential hydraulic strategies across provenances. Accordingly, this study highlights the limitations of interpretations based solely on physiological indicators and provides a more mechanistic understanding of the metabolic processes driving hydraulic adjustment under drought conditions. In summary, the present study not only extends the conceptual framework proposed by Gao et al.^[25]^, but also offers valuable new insights into the spatial and physiological variability of hydraulic strategies, contributing to more effective drought management and advancing our understanding of plant resilience under climate change.

## Conclusions

This study investigated the hydraulic strategies of *C. lanceolata* from provenances with contrasting historical drought regimes under controlled drought conditions. Using the TVDI, historical drought intensity was linked to provenance-specific physiological responses in greenhouse experiments. Seedlings from arid regions maintained stable water status under drought through conservative hydraulic strategies, whereas those from humid regions displayed greater physiological plasticity and prioritized growth. Distinct metabolic adjustments supported these strategies, reflecting trade-offs between water conservation and resource acquisition. Importantly, these results highlight the ecological significance of hydraulic diversity: conservative water-use patterns enhance survival in prolonged drought, while flexible strategies promote growth in wetter environments. Drought-adapted provenances represent valuable genetic resources for afforestation in water-limited areas, and their associated metabolic pathways may serve as markers for selecting drought-tolerant genotypes. Overall, the present findings link long-term adaptation to specific hydraulic strategy and provide guidance for sustainable management of *C. lanceolata* under future climate change.

## SUPPLEMENTARY DATA

Supplementary data to this article can be found online.

## Data Availability

The datasets generated during and/or analyzed during the current study are available from the corresponding author upon reasonable request. The supplementary materials can be found at: https://doi.org/10.6084/m9.figshare.30174892.v1.
