# Supplementary material for: Hydraulic strategies of Cunninghamia lanceolata under drought are shaped by native drought conditions
Source: For Res (Fayettev). 2025 Dec 31;5:e031. doi: 10.48130/forres-0025-0031 (PMC12979179; doi:10.48130/forres-0025-0031)
Supplement: Supplementary file 1 — Supplementary data to this article can be found online. [file forres-0025-0031-Supplementary.zip › 10.48130_forres-0025-0031-Suppl-File1.docx]

**Supplementary File1. Hydraulic strategies of *Cunninghamia lanceolata* under drought are shaped by native drought conditions**

**1. Remote-Sensing data sets**

**Moderate Resolution Imaging Spectroradiometer (MODIS), a component of the National Aeronautics and Space Administration Earth (NASA) Observation System, generates highly reliable datasets extensively recognized and utilized^[1.2]^. MOD13Q1 product offers vegetation indices, including the Normalized Difference Vegetation Index (NDVI) and the Enhanced Vegetation Index (EVI). The data are composited every 16 days at a spatial resolution of 250 m. Compared to NDVI, EVI reduces canopy background noise and retains sensitivity in high vegetation density conditions^[3]^. In this study, monthly EVI data was processed to extract the maximum value for each period. MOD11A2 provides Land Surface Temperature (LST) data at a 1 km resolution. The average daytime LST was extracted monthly, corresponding to the MODIS Terra satellite’s overpass time of approximately 10:30local time.**

**The datasets cover the period from January 2000 through December 2020 and were resampled to a unified 1 km resolution. Quality control relied on the quality assurance flags associated with each dataset. Cloud and snow contaminated pixels were excluded and bilinear interpolation was applied to fill the gaps. The Savitzky-Golay filter method^[4]^ was applied to further reduce residual noise in the EVI and LST time series. All pre-processing and analysis were performed on the Google Earth Engine (GEE) platform.**

The elevation data used in this study were obtained from NASA's NASADEM Digital Elevation product, which features a spatial resolution of 30 m. The elevation data for the study area were extracted using the GEE platform and resampled at a spatial resolution of 1 km. The resampled elevation was used to correct the LST data and calculate the TVDI.

Soil moisture data were obtained from the Science Data Bank, derived from measurements at 1,471 soil moisture monitoring stations throughout China. After strict quality control and data transformation, the data set includes soil moisture information for 732 stations at five depths (10, 20, 50, 70, and 100 cm)^[5]^. For the study area, data from 36 meteorological stations were selected for analysis. As the China Meteorological Administration uses 20 cm soil moisture as the standard for drought classification^[6]^, the 20 cm data were used to validate the reliability of the TVDI results.

**2. Remote sensing data analysis**

Due to the significant influence of air temperature and atmospheric turbulence, the LST varies greatly with elevation^[7]^. The study area exhibits severe topographic heterogeneity. To eliminate the effect of elevation on LST, a correction is applied to the surface temperature data as shown in equation (1):

$$\begin{aligned} T_{S}=T+a*H \#\left( 1 \right) \end{aligned}$$

where *T_S_* is the corrected LST (°C), *T* is the original LST retrieved from MOD11A2 (°C), *a* is the correction coefficient set at 0.006^[8]^ and *H* is the elevation derived from the elevation (m). This correction was performed simultaneously during the generation of monthly LST data, ensuring consistent and accurate results throughout the study period.

Soil moisture is closely related to LST and vegetation growth, typically exhibiting a near-linear correlation^[9]^. Under wet conditions, adequate soil moisture promotes vigorous growth of vegetation, which in turn reduces LST through evapotranspiration. In contrast, during drought conditions, insufficient soil moisture limits vegetation growth, leading to an increase in LST. Building on this relationship, Sandholt et al.^[10]^ proposed the simplified drought index, TVDI, derived by constructing a two-dimensional feature space using LST and NDVI. The distribution of data points within this feature space reflects variations in the soil moisture gradient, allowing a quantitative assessment of the water stress experienced by vegetation. The TVDI was calculated according to equation (2):

$$\begin{aligned} TVDI=\frac{T_{S}-T_{min}}{T_{max}-T_{min}} \#\left( 2 \right) \end{aligned}$$

where *T_S_* is the LST of any pixel, *T_min_* is the lower boundary (wet edge) of the LST-EVI, and *T_max_* is the upper boundary (dry edge). TVDI values range from 0 to 1, with higher values indicating more severe drought and lower values reflecting wetter conditions. The wet and dry edges were determined by linear regression and expressed as:

$$\begin{aligned} T_{min}=a_{1}+b_{1}*EVI \#\left( 3 \right) \end{aligned}$$

$$\begin{aligned} T_{max}=a_{2}+b_{2}*EVI \#\left( 4 \right) \end{aligned}$$

where *a_1_* and *a_2_* are intercepts, and *b_1_* and *b_2_* are slopes of the wet (3) and dry (4) edge equations, respectively. These parameters were derived using least squares regression by extracting the minimum and maximum LST values within EVI intervals of 0.01 across the LST–EVI feature space.

To further understand the temporal trends in drought conditions in the study area, we applied the Theil-Sen median method to analyze trend changes in the monthly average TVDI values and used the Mann-Kendall nonparametric test to assess the significance of these trends^[11]^. The calculation process was implemented using Python (version 3.10) and the graphics were generated using ArcGIS 10.8 (Esri, Redlands, CA, USA).

The Theil-Sen median method is a robust nonparametric statistical technique that makes no specific assumptions about data distribution and is resistant to outliers. It provides more reliable slope estimates than ordinary least squares regression. This method is widely used in remote sensing time series data analysis:

$$\begin{aligned} Sen=median\left( \frac{{TVDI}_{i}-{TVDI}_{j}}{i-j} \right) \#\left( 5 \right) \end{aligned}$$

**where *Sen* represents the slope of the trend, with positive values indicating an upward trend and negative values indicating a downward trend; *i* and *j* represent months, with *i*>*j*; *TVDI_i_*and *TVDI_j_*are the TVDI values for months *i and j*,respectively.**

To assess the statistical significance of the trends, this study applied the Mann-Kendall test. The Mann-Kendall test is a nonparametric method used to detect monotonic trends in time-series data without assuming a specific distribution.

$$\begin{aligned} S=\sum_{k=1}^{n-1} \sum_{j=k+1}^{m} \mathrm{sign}\left( {TVDI}_{j}-{TVDI}_{k} \right)\#\left( 6 \right) \end{aligned}$$

where ***n*** represents the length of the time series; ***k*** and ***j*** denote months; ***TVDI_k_*** and ***TVDI_j_***represent the TVDI values for months ***k*** and ***j***, respectively. The **sign** function is defined as:

$$\begin{aligned} sign\left( {TVDI}_{j}-{TVDI}_{k} \right)=\left\{ \begin{aligned} 1 if {TVDI}_{j}>{TVDI}_{k} \\ 0 if {TVDI}_{j}={TVDI}_{k} \\ -1 if {TVDI}_{j}<{TVDI}_{k} \end{aligned} \right.\#\left( 7 \right) \end{aligned}$$

**3. Measurement ofleaf physiological indices**

**3.1 Measurement of biomass-related indicators**

Biomass-related traits, including leaf weight (LW), stem weight (SW), aboveground biomass (AB), root weight (RW), total biomass (TW), and the root-to-shoot ratio (RST), were determined at the conclusion of the drought treatment period. To ensure accuracy in biomass quantification, seedlings were gently excavated in their entirety, with special care taken to preserve the integrity of the root system. Adhering soil was carefully removed by hand, after which the roots were briefly rinsed with distilled water to eliminate residual particles and subsequently blotted dry using non-woven, lint-free fabric. Following cleaning, each plant was separated into three anatomical components: needles, stems (defined as the portion of the shoot located at least 2 cm above the root–shoot junction), and roots. All tissues were then subjected to oven-drying at 80 °C for a minimum of 72 hours, or until a constant mass was achieved, thereby ensuring the complete removal of moisture. The dry weight of each component was measured using an analytical balance with a precision of 0.1 mg. Leaf weight (LW) was defined as the total dry mass of needles per individual seedling, while stem weight (SW) referred to the dry mass of the shoot portion above the root collar. Aboveground biomass (AB) was calculated as the sum of LW and SW, and root weight (RW) was obtained by weighing the entire dried root system. Total biomass (TW) was determined as the sum of aboveground and belowground biomass (AB + RW). The root-to-shoot ratio (RST), representing the relative allocation of biomass belowground versus aboveground, was calculated by dividing RW by AB (RST = RW / AB).

**3.2 Measurement of photosynthetic parameters**

Net photosynthesis rate (Pn), stomatal conductance (Gs), and transpiration rate (Tr) were determined using a portable open gas exchange system (LI-6400XT; LI-COR Biosciences, Lincoln, NE, USA). Measurements were conducted under a saturated photosynthetic photon flux density of 1200 µmol m⁻² s⁻¹, which was optimized based on preliminary trials, with chamber conditions set to a needle temperature of 28 ± 2 °C and a constant CO₂ concentration of 450 µmol mol⁻¹, closely reflecting ambient atmospheric conditions between 08:00 and 11:30 a.m. A CO₂ gas cylinder supplied a stable CO₂ flow to ensure consistency. Prior to measurement, seedlings were illuminated under assay light for 5–10 minutes to allow full induction of photosynthetic activity. Measurements were performed on intact needle fascicles, and once gas exchange reached a steady state, Pn, Gs and Tr were recorded. After measurement, the same needles were scanned with a flatbed scanner, and the projected needle area was quantified using ImageJ software (National Institutes of Health, USA) to normalize gas exchange values. Instantaneous water use efficiency (WUEi) was calculated as the ratio of net photosynthesis to transpiration (WUEi = Pn/Tr), following the approach of Farquhar et al.^[12]^.

**3.3 Measurement of antioxidant enzyme activities**

The content of hydrogen peroxide (H₂O₂), as well as the enzymatic activities of peroxidase (POD) and superoxide dismutase (SOD), were determined using commercial assay kits following the manufacturer’s instructions (Beijing Solarbio Science & Technology Co., Ltd). Briefly, tissue homogenates were prepared and the supernatants reacted with the corresponding reagents: H₂O₂ content was quantified via a colorimetric reaction with the kit’s chromogenic substrate under controlled conditions, and absorbance was measured at 415 nm. POD activity was assessed by monitoring the rate of substrate oxidation, indicated by absorbance changes at 470 nm, while SOD activity was determined based on its ability to inhibit the reduction of nitroblue tetrazolium, with absorbance read at 560 nm to quantify enzyme activity. Ascorbate peroxidase (APX) activity was measured colorimetrically following the method of Nakano and Asada^[13]^with slight modifications. Approximately 0.1 g of frozen needle tissue was homogenized in 2 mL of ice-cold 50 mM potassium phosphate buffer (pH 7.0) containing 1 mM ascorbic acid and 5.6 mM H₂O₂. The homogenate was centrifuged, and the absorbance of the supernatant was recorded at 290 nm to assess APX activity.

**3.4 Measurement of osmotic adjustment substances**

Soluble sugars, fructose, and sucrose were extracted from oven-dried needle samples using 80% (v/v) ethanol at 80 °C for 30 minutes in a water bath, followed by centrifugation at 8,000 g for 5 minutes^[14]^. The total soluble sugar content was quantified colorimetrically at 625 nm using the anthrone method based on 50 mg of the dried sample^[14]^. Fructose and sucrose concentrations were further analyzed by high-performance liquid chromatography (HPLC) using a Thermo Scientific™ UltiMate™ 3000 system equipped with a C18 chromatographic column (Xtimate, 1.8 µm, 2.1 × 100 mm). The column was maintained at 75 °C and operated isocratically with water as the mobile phase at a flow rate of 0.4 mL min⁻¹. Identification and quantification of sugars were performed by comparing retention times and peak areas against known sugar standards. Following ethanol extraction, starch content was determined colorimetrically at 620 nm from the residual pellet according to Dubois et al.^[15]^. Total non-structural carbohydrates (NSCs) were calculated as the sum of soluble sugars and starch content^[16]^. Proline concentration was measured using the sulfosalicylic acid method described by Bates et al.^[17]^.

**4. Measurement of leaf metabolites**

Metabolites in the leaves of *C. lanceolata* subjected to different treatments were quantified using a broad-targeted metabolomics approach. The frozen dried leaf samples were ground into a fine powder using a ball mill (MM 400, Retsch, SCIENTZ, China) at 30 Hz for 1.5 min. A 100 mg quantity of the powdered sample was weighed and dissolved in 0.6 ml of 70% methanol extraction solution, followed by incubation at 4°C for 12 h. The resulting solution was centrifuged at 10,000 rpm for 10 min. The supernatant was filtered through a 0.22 μm pore-size membrane filter prior to UPLC-MS/MS analysis.

Leaf metabolites were analyzed by UPLC (Shim-pack UFLC SHIMADZU CBM30A, https://www.shimadzu.com.cn/) and MS/MS (Applied Biosystems 4500 QTRAP, http://www.appliedbiosystems.com.cn/). Chromatographic separation was performed using an Agilent SB-C18 column (100 × 2.1 mm, 1.8 μm, Waters Corporation, Taunton, Massachusetts, USA), with the temperature maintained at 40°C. An injection volume of 4 μl was used and a binary solvent system consisting of solvent A: 0.1% formic acid + 99.99% ultrapure water (v/v) and solvent B: 100% acetonitrile was used. The elution was carried out at a flow rate of 0.35 ml/min under the following gradient conditions: B increased from 5% to 95% over the first 11 min, held at 95% for another minute, followed by a 3 min reequilibration period. Mass spectrometry conditions included: peak detection was performed with an electrospray ionization source temperature of 550°C, an ion spray voltage of 5500 V, a curtain gas of 30 psi, and a collision-activated dissociation parameter set to high. In the triple quadrupole, each pair of ions was scanned and detected according to the optimized decluttering potential and collision energy (Chen et al., 2013). Data were filtered, aligned and calculated using Analyst (version 1.6.3) (AB SCIEX, Framingham, Massachusetts, USA) and MultiaQuant (SCIEX, MA, USA). To generate a matrix with less biased and redundant data, peaks caused by different isotopes, fragmentation of the source, K^+^, Na^+^, NH4^+^ and dimerization were checked and removed. The metabolites in the samples were identified by comparing accurate m/z values, retention times, and fragmentation patterns in the self-built MWDB database (Metware Biotechnology Co., Ltd., Wuhan, China).

**References**

1. Son NT, Chen CF, Chen CR, Chang LY, Minh VQ. 2012. Monitoring agricultural drought in the Lower Mekong Basin using MODIS NDVI and land surface temperature data. International Journal of Applied Earth Observation 18:417–27. doi: 10.1016/j.jag.2012.03.014
2. Khosravi Y, Homayouni S, Ouarda TBMJ. 2024. Spatio-temporal evaluation of MODIS temperature vegetation dryness index in the Middle East. Ecological Informatics 84:102894. doi: 10.1016/j.ecoinf.2024.102894
3. Rossi E, Rogan J, Schneider L. 2013. Mapping forest damage in northern Nicaragua after Hurricane Felix (2007) using MODIS enhanced vegetation index data. GIScience & Remote Sensing 50:385–99. doi: 10.1080/15481603.2013.820066
4. Savitzky A, Golay MJE. 1964. Smoothing and differentiation of data by simplified least squares procedures. Analytical Chemistry 36:1627–39. doi: 10.1021/ac60214a047
5. Wang AH, Shi XL. 2021. A multilayer soil moisture dataset based on the gravimetric method in China [DS/OL]. Science Data Bank. doi: 10.11922/sciencedb.00539
6. Martínez-Fernández J, González-Zamora Á, Sánchez N, Gumuzzio A. 2015. A soil water based index as a suitable agricultural drought indicator. Journal of Hydrology 522:265–73. doi: 10.1016/j.jhydrol.2014.12.051
7. Zhao T, Guo W, Fu C. 2008. Calibrating and evaluating reanalysis surface temperature error by topographic correction. Journal of Climate 21:1440–46. doi: 10.1175/2007jcli1463.1
8. Li C, Adu B, Wu J, Qin G, Li H, et al. 2022. Spatial and temporal variations of drought in Sichuan Province from 2001 to 2020 based on modified temperature vegetation dryness index (TVDI). Ecological Indicators 139:108883. doi: 10.1016/j.ecolind.2022.108883
9. Gao Z, Gao W, Chang NB. 2011. Integrating temperature vegetation dryness index (TVDI) and regional water stress index (RWSI) for drought assessment with the aid of LANDSAT TM/ETM+ images. International Journal of Applied Earth Observation 13:495–503. doi: 10.1016/j.jag.2010.10.005
10. Sandholt I, Rasmussen K, Andersen J. 2002. A simple interpretation of the surface temperature/vegetation index space for assessment of surface moisture status. Remote Sensing of Environment 79:213–24. doi: 10.1016/s0034-4257(01)00274-7
11. Yan F, Zhang Y, Wang X, Xu Z, Liang Y, et al. 2025. Characteristics of spatial and temporal non-stationarity of groundwater storage in different basins of China and its driving mechanisms. Journal of Hydrology 655:132882. doi: 10.1016/j.jhydrol.2025.132882
12. Farquhar GD, Ehleringer JR, Hubick KT. 1989. Carbon isotope discrimination and photosynthesis. Annual Review of Plant Biology 40:503–37. doi: 10.1146/annurev.pp.40.060189.002443
13. Nakano Y, Asada K. 1981. Hydrogen peroxide is scavenged by ascorbate-specific peroxidase in spinach chloroplasts. Plant and Cell Physiology 22:867–80. doi: 10.1093/oxfordjournals.pcp.a076232
14. Liao J, Song H, Tang D, Zhang S. 2019. Sexually differential tolerance to water deficiency of Salix paraplesia—A female‐biased alpine willow. Ecology and Evolution 9:8450–64. doi: 10.1002/ece3.5175
15. Dubois M, Gilles KA, Hamilton JK, Rebers PA, Smith F. 1956. Colorimetric method for determination of sugars and related substances. Analytical Chemistry 28:350–56.
16. Jordan MO, Vercambre G, Gomez L, Pages L. 2013. The early spring N uptake of young peach trees (Prunus persica) is affected by past and current fertilizations and levels of C and N stores. Tree Physiology 34:61–72. doi: 10.1093/treephys/tpt109
17. Bates LS, Waldren RP, Teare ID. 1973. Rapid determination of free proline for water-stress studies. Plant and Soil 39:205–07.
